# Authentic roles matter: how in situ simulation influences fidelity, psychological safety, and learning transfer

**DOI:** 10.1186/s41077-026-00449-4

**Published:** 2026-06-08

**Authors:** Kenneth Boe Krarup, Alexandra Claire McKenzie, Ayham Al-Masri, Lotte Abildgren

**Affiliations:** 1 Department of anesthesia and intensive care, Odense University hospital, Odense, Denmark; 2 Department of anesthesia and intensive care, Esbjerg Sygehus, Denmark; 3 The Innovation and Clinical Research Department, Hospital of South Jutland, Aabenraa, Denmark; 4 Department of Regional Health Research, The University of Southern Denmark, Odense, Denmark; 5 Centre for Research in Patient Communication, Odense University Hospital, Odense, Denmark; 6 Department of Clinical Research, University of Southern Denmark, Odense, Denmark; 7 SimC - Simulation Centre, Odense University Hospital, Odense, Denmark

**Keywords:** In situ simulation, Cross-training, Fidelity, Psychological safety, Transfer, Qualitative

## Abstract

**Background:**

In situ simulation (ISS) enables healthcare teams to test systems, identify safety threats, and strengthen interprofessional practice. Compared with off-site simulation (OSS), ISS may promote greater fidelity and transfer of learning. Cross-training, where participants adopt roles outside their profession, is widely used in healthcare education but remains controversial, with little evidence on how participants perceive its impact.

**Methods:**

We conducted a qualitative study in a Danish teaching hospital intensive care unit, using ethnographic observations, field notes, and semi-structured interviews. Sixteen interviews with physicians and nurses, supported by audio recordings of debriefings, were thematically analysed using a phenomenological-hermeneutic approach with consensus coding.

**Results:**

Four themes were identified: fidelity, psychological safety, transfer, and cross-training. Participants described fidelity as multidimensional, including environmental, team, scenario, time, and resource aspects. ISS was perceived as more realistic and immersive than OSS, supporting deeper engagement and learning. PS was enhanced by familiarity with colleagues and environment, limited observation, and absence of cross-training. Facilitator competence at facilitating politely and creating room for all participants to speak up strongly influenced PS. ISS was viewed as promoting greater transfer of technical and social-cognitive skills to clinical practice, with some learning leading to organisational change. Cross-training was met with mixed reactions. Most participants, especially junior staff, preferred training in authentic roles, citing negative effects on PS and learning. More experienced clinicians occasionally valued cross-training for its reflective potential, though often only in less advanced roles.

**Conclusion:**

In situ simulation promotes learning by aligning fidelity, psychological safety, and transfer within participants’ authentic clinical roles and environments. Cross-training was frequently perceived as undermining both learning and psychological safety, particularly among less experienced staff, suggesting a misalignment between role substitution and learners’ developmental needs. Careful scenario design emphasising authentic roles, realistic fidelity, and skilled facilitation may optimise simulation learning outcome, Cross-training should be applied selectively, with explicit attention to learners’ experience levels, role authenticity, and potential impacts on psychological safety.

## Background

This article reports an ethnographic, interview-based case study on in situ simulation (ISS) and healthcare professionals' experiences with cross-training (CT).

It is known that ISS offers an opportunity to test medical organisations [1] and possibly reveal latent safety threats in the clinical setting [2, 3]. Research shows that implementing ISS-based training programmes improves timing in trauma care [4] and can help facilitate cultural changes, increasing patient safety [5]. ISS offers a higher degree of environmental fidelity. Brazil et al. introduced the concept of translational simulation in 2017 and further explored it in 2024 [6, 7]. Translational simulation is to be understood as how simulation can be used as a tool to facilitate a process from one point to another, within the organisation or the individual [6, 7]. The goal of translational simulation is to enhance the understanding of simulation, shifting the focus away from the training itself. Therefore, ISS is to be considered more as a tool to the goal than the goal itself. However, knowledge of how ISS is perceived by participants to gain learning on an individual basis seems to be sparse.

Volpe et al. [8] define cross-training as a training strategy where *“each team member is trained on the tasks, duties, and responsibilities of his or her fellow team members” (p. 87*). For instance, a doctor acts as a nurse in the training session. Within the specialist training in anaesthesia and intensive care in Denmark, interprofessional off-site simulation (OSS) and cross-training are commonly used for practical and logistical reasons. Traditionally, healthcare education has been divided into silos [9], which creates challenges when training for daily work life [8]. When using cross-training, we have observed that roles are often portrayed in a stereotyped or caricatured ways, creating a false perception of the clinical environment. Interprofessional teamwork is a key concept in clinical practice [10]. A debate about the value of cross-training persisted in our faculty. Cross-training has previously been perceived to enhance team performance [11, 12]. However, Sørensen et al. [10] found that participants had a high preference for training in authentic roles. It was not clarified who or why they had this preference. Thus, knowledge of cross-training remains sparse [11–14], particularly regarding its application in healthcare simulation.

We have identified a knowledge gap about how learners' perception of both cross-training and ISS in healthcare.

## Aim

This study aims to explore the factors in ISS that influence learning and how participants in ISS perceive cross-training.

## Methods

### Study design

A qualitative, phenomenological-hermeneutic methodology, combined with an ethnographic study, examined how participants experienced cross-training, how ISS facilitated learning, and how it influenced learning outcomes. The STROBE statements are used as reporting guidelines in this article [15].

### Ethnography

Based on the theory of participant observation [16], organisational ethnography [17], and the guidelines on interview-based qualitative research [18], the author (KBK) collected ethnographic data and conducted semi-structured interviews of critical care professionals. KBK participated as an ethnographic observer during the ISS and served as the ethnographic observer during the subsequent debriefings. Based on the ethnographic data, an interview guide was created. The data compiles field notes and audio recordings from the debriefings and interviews. When needed, participants were contacted for confirmation of the interpretation of the interviews [19].

### In situ simulation

The ISS occurred 3–4 times a year. Scenarios were developed by KBK and addressed everyday clinical situations. ISS had been running for several years with different expert facilitators (facilitator course equivalent to EUSim levels 1 and 2). Participation was not mandatory. The patient simulator was a HAL S3201 [20].

An ISS day consisted of three simulation-training sessions; each participant engaged in only one simulation per day. Each session lasted 90 minutes, divided into: a five-minute pre-brief introducing the patient simulator and rules of engagement, approximately 30 minutes for the simulation, approximately 30 minutes for the simulation and 45 minutes for debriefing. In the 3 months following the training sessions, participants were contacted to join a semi-structured interview.

### Settings

The intensive care unit consists of eight beds at a teaching hospital (809 beds).

### Participants

All participants (intensive care nurses, junior and senior anaesthesiologist) had clinical work on the day of the simulation. They were informed about their ISS timeslot roughly a week before the simulation. If any scheduled participants were absent on the day of the simulation, a new participant was appointed on the day. KBK and the lead of the ISS programme appointed the participating doctors. The nurse facilitators appointed the nurse participants. Two doctors (one junior and one senior) and two or three nurses (from all levels) participated in each scenario.

### Ethics

All participants were informed of the study. Written consent was provided. Participation in the study was voluntary.

The Danish Regional Committee on Health Science and Ethics (S-20222000-71) and The King's College ethical committee (LRU/DP-21/22–28543) approved the study.

### Data analysis

Field notes were handwritten during the observations. All interviews were transcribed and analysed for themes and codes [21, 22]. Audio files of debriefings were reviewed. To ensure consistency and improve the quality of the interpretation, two authors (LA and KBK) consensus-coded the data material [23].

## Results

The data consist of field notes, recordings from four debriefings (2h, 57 min, 37sec), and audio recordings of semi-structured interviews (4h, 31 min, 27sec). Interviews were conducted from September to December 2022. They ranged from 12:11 minutes to 28:45 minutes. The 30 planned interviews were reduced to 16 due to data saturation (Table 1). Interviewees were selected using convenience sampling to ensure representation from all professions involved in the simulation. Audio recordings of the debriefings and field notes were reviewed and interpreted to identify codes and themes. The analysis resulted in four key themes: 1) fidelity; 2) psychological safety; 3) transfer; and 4) cross-training. Each has several subthemes, shown in Table 2.

**Table 1:** Participants included in the study

Participants			
Number	16	Male Female	4 12
Age	Average 45	25–40 years +41 years	8 8
Occupation	Nurse Junior doctors Senior doctors	6 7 3	
postgraduate work experience	Average 14.5 years	0–5 years 6–15 years +16 years	7 3 6
OSS Experience	Average 13,1 days	0–5 daysc +6 days	9 7
ISS EXPERIENCE	Average 13,1 days	0–5 days +6 days	12 4

**Table 2:** Example of the analytical process, from what was said, heard, and seen to the emerged themes and subthemes

Fidelity	Debriefing*	“Debriefing is where the learning takes place.” “Generic roles in debriefing enhances learning.” “Debriefing becomes more realistic leading to learning.” “Debriefing in your role is important.”
	Communication skill training	“Realism is better for communication, which makes learning better.” “Communication learning is better ISS.” “Communication learning is about getting to know the group better.”
	Ability to Immerse into the scenario	“You will need lesser efforts to immerse into a scenario during ISS.” “It is easier to immerse in a scenario during ISS.” “I tend to take it less seriously when it is not realistic, which is more likely during ISS.”
	Time frame	“That ISS frequently is training in max 2 hours enhances learning.”
	Resources realism	“That resource reflects daily resources enhances learning.”
	Scenario realism	“That scenario is realistic and enhances learning.” “Scenario frequent unrealistic during OSS = negative for learning.”
	Team realism	“Realism in teams and environment enhances learning.” “That team reflects normal team members, enhances realism, and thereby learning” “Being in generic roles enhances learning.” “ISS is like reality in regards to teams.”
	Time realism	“Time realism creates a realistic feeling of stress.” “Time realism contributes to learning.” “The Time factor contributes to learning.” “Time factor contributes to the feeling of good stress.”
Psychological safety	Psychological safety	“Stress level higher because team members were not known to participants.” “Psychological safety because the environment is known to enhance learning”
	Stress/Anxiety	“Higher level of stress OSS” “Stress level is the same as ISS and OSS.” “The feeling of stress during ISS resembles the feeling you have in reality which is good.”
	Motivation	“Being Motivated to learn does not influence learning.”
	Negative influence on learning	“ISS the debrief tends to be less honest because you know the Colleagues” “Nothing negative about ISS” “Many observers => makes you more insecure =>decrease learning”
	Instructors’ role	“If addressing participants in a bad way decreased learning.” “Both fellow participants and instructors need to be polite for learning to take place.” “The instructor's ability to do both sim and debrief is more important than ISS or OSS”
Transfer	Organisational learning	“Organizational learning enhanced during ISS.” ”ISS enhances organizational understanding.”
	Transfer	“The more realistic, the higher the transfer.” “ISS leads to better transfer.” “Higher level of transfer when doing ISS”
Cross-training	Cross-training	“Nothing feels negative about cross-training.” “Fears to get a role above own level of competence” “Limited learning from Cross training. The only thing might be how hard leadership, for instance, could be.” “Cross training takes away focus from learning.”

*For pragmatic reasons, debriefing was placed under fidelity. While it could also have been represented in other sections, we did not consider it to warrant a separate section

### Fidelity

The theme of fidelity is characterised by how realistic the participants perceived the learning environment.

The theme emerges across data. Participants consistently reported a high degree of immersion during in situ simulation. Participants identified fidelity as a key factor in facilitating learning during ISS. Multiple elements were revealed and were categorised as follows: time fidelity, environmental fidelity, scenario fidelity, team fidelity, and resource fidelity.

#### Environmental fidelity

A junior doctor and a junior nurse narrate that *“There is an extra layer of my brain that I do not need to use to visualise the surroundings” (I) and “I will take it more seriously during ISS” (I).* Running the scenario in their clinical enviornment might facilitate participants engagement in role play during ISS. This leads us to say that the environment fidelity influences the way the participants interact within the scenario. Several participants mentioned that unrealistic time allocation for tasks negatively affected their learning. E.g., some reported that being informed they had successfully inserted an intravenous catheter on a baby (simulator) without performing the task hindered their ability to engage with the rest of the simulation. One senior doctor experienced this in both ISS and OSS but observed that time fidelity is more likely to be achieved during ISS. This suggests that ISS should improve its ability to replicate time fidelity, thereby leading to a higher degree of engagement.

Many participants consider environmental fidelity essential for effective learning in this study. Nine participants report that environmental fidelity significantly impacts their learning experience. Two participants mentioned it as insignificant. One participant suggested that achieving environmental fidelity in OSS is possible but less probable. Furthermore, mistakes made during OSS might be attributed to an unrealistic environment, thus impeding learning. This observation was supported by both the debriefing audio files and field notes. Our interpretation is that environmental fidelity matters and is harder to replicate in OSS. Performing a simulation in a less realistic environment can lead to an excuse for mistakes, thereby hindering learning. Team composition and scenario fidelity is perceived to be vital for learning during ISS. Exemplified by: *“Having a team that reflects normal* [having your role] *team members enhance fidelity, thereby facilitating learning” (I).* Also, scenario fidelity is perceived to be vital in ISS. *“When the scenario closely mirrors your usual reality, you become more confident” (I).* This theme emerges significantly in the audio files and field notes. It appears that when running ISS, the faculty manage, to a high degree, to compose the simulation team and run the scenarios that mirror the daily clinical practice. A participant mentions that unrealistic scenarios might even hinder the learning process. *“I become obstructive if the scenario is unrealistic, which is detrimental to learning” (I).*

### Psychological safety

This theme is characterised by how psychologically safe (PS) the participants feel during the simulation and debriefing. PS affects how much a person speaks up, expresses their opinions, and takes risks during the simulation and debriefing [24]. Many participants express that they believe ISS has a positive impact on PS. E.g., PS is perceived as higher when the participants are familiar with each other and with the simulation environment, citing: “*you know the ones you simulate with and the environment” (I),* they maintain their professional role in the simulation *“you get to play your own role”(I),* and if there is no observer in the simulation setting *“there are no or few observers”(I).* Data from interviews and observations suggest that prior familiarity with the team can also affect PS negatively, as existing intercollegiate relationships influence training. It is unclear whether a group consistently perceives prior knowledge of the team positively or negatively. The factors can be divided into two subthemes: internal and external. Internal factors include collegial relationships and the national culture of power distance in the workplace, whereas external factors include prior knowledge of the environment, the absence of role-playing, and the absence of observers. We believe that the bilateral influence of previous knowledge on the team may mirror the daily PS in the department, which vary from person to person. These factors could influence learning both positively and negatively.

Participants highlight the crucial role of the facilitator's influence on PS. The facilitator's competence is vital during the scenarios and debriefing. A senior nurse stated, *"Politeness from both the instructor and participants is essential for learning"* (*I*). Adding to that, another participant states that the rescence of a competent facilitator outweighs the type of simulation, whether it is ISS or OSS. Two participants, who are trained simulation facilitators, highlight the importance of facilitator competency in education. Field notes indicate that when the debriefer is skilled at creating PS during the debrief, they contribute to the participants' engagement in the discussion. Thus, the optimal PS learning environment is to some extent dependent on the facilitator’s ability to foster it.

Several participants highlight the importance of stress during simulation training. Two out of three senior doctors express their experiences on stress levels, noting their positive impact on their learning. This is evident in the following statements: *“Being under time pressure (during the simulation) would teach you how to handle stress” (I)* and *“the feeling of being a bit insecure is good for learning” (I).* This emphasises that a certain degree of stress is inevitable and can enhance learning in some individuals, making it an essential component of their learning objectives. Based on the study data, it does not appear to have a negative influence on PS.

### Transfer

This theme describes how the participants apply what they learnt from the simulation activities to their daily job. 81% agree that ISS increases transfer compared to OSS. Only one participant believes that the scenario is more crucial for transfer than the setting where the simulation takes place. *“Transfer from doing OSS and ISS is just as likely, but there are different things you take with you to the clinical setting” (I).* Two participants noted that they had experienced learning from simulations applied directly to the clinical setting. Several interviews revealed that factors influencing transfer include fidelity and the debriefing. From the audio files of debriefings and field notes, it becomes evident that several participants have examples of both technical and non-technical (i.e. SCOPE) they acquired from ISS and subsequently directly transferred into their clinical work. Observing the transfer of SCOPE from the simulation to daily clinical work was not as straightforward as for technical skills. Our data indicates that participants perceive transfer of both SCOPE, and technicals skills to be more likely when running an ISS rather than OSS.

In addition, safety concerns from the debriefing and observation during simulation were reported by faculty to departmental administration to improve patient safety. For instance, the equipment for frontal neck access in the emergency tracheostomy kit was changed after ISS revealed how hard it was to open the package.

### Cross-training

This theme elaborates on how learners perceive cross-training. In this study, we found a mixed attitude towards cross-training and a strong preference for training in one's professional role. Certain participants reported: “Cross*-training does not lead to a better understanding of other team members*” (I) and *“no learning from cross-training*” (*I*); however, some participants argue that cross-training could lead to meaningful learning. “*Cross-training leads to better understanding of your communication when in your own role later” (I).* Field notes revealed that some participants who are assigned to cross-training roles tend to act comically in their assigned roles. A tendency in this direction is evident in our interviews as well. However, the participants who are clinically very experienced, tend to have a positive attitude towards cross-training. “*Cross-training contributes positively to your learning experience” (I*). In contrast, younger participants report that cross-training has a negative impact on their PS during OSS. “*OSS gives the sense of no PS because of cross-training” (I).* If cross-training is used, it appears that participants prefer to cross-train in roles, where their clinical expertise is not required, such as porters. Junior nurses may learn valuable skills when acting as porters, and junior doctors often find cross-training as nurses acceptable. This might be due to a high degree of their self-efficacy in their professional role as they advance in their career. Field notes indicate that the faculty, in general, view cross-training positively, because they, possibly, have a broader perspective on learning and believe that self-reflection enhances personal understanding, which can lead to stronger leadership and teamwork within the team.

## Discussion

Our findings reveal the perceived value of fidelity and suggest that transfer and PS are experienced at higher levels in ISS compared to OSS. The study also provides new insights into participants’ perceptions of cross-training in simulation training. We aimed to pinpoint factors influencing learning in ISS and to reveal participants' perceptions of cross-training. The following section will compare our results with those in the existing literature.

### Fidelity

We found that fidelity influences learning in different ways. This phenomenon is supported by several researchers [25–27]. Our findings indicate that the environment and equipment are essential for learning. Our participants experienced training in the same environment in which they conduct their clinical work, which means that the transfer process improves. Paige et al.'s [27] findings support that fidelity need to match learning goals. [10, 27]. However, Sørensen et al. [10] conducted a study involving focus group interviews with participants in both OSS and ISS. Participants had less preference for the simulation setting being OSS or ISS. This contradicts our findings, which show that prior knowledge of the environment and equipment enhances learning. Sørensen et al. studied obstetricians, nurses and midwives running ISS. In our study, we interviewed anaesthesiologist (juniors and seniors), and ITU nurses. This could explain the difference in perception of the importance of fidelity and indicate that context influences the perception. Our study and Sørensen et al agree that participants in simulation training prefer to train in their habitual role. Sørensen et al. did not clarify whether learners also preferred the rest of the team to be in their generic professional roles.

Moreover, we found that scenarios being representative of participants' everyday work life is essential for learning; this correlates with the findings of Dieckmann et al.'s [25], in which the perception of conceptual fidelity is described as how much the simulation can resemble a real-life case. This highlights an increased need for focus on scenario fidelity. We found that familiarity with the environment, team, and location enhances participants' immersion and supports learning in the simulation. Several other authors have

contributed to this [26–30]. E.g., Norman et al. [26], who made a review of the literature comparing high vs low fidelity, and provide an overview of five different methods for assessing fidelity settings. 20% of the studies show that trainees have a higher degree of knowledge retention in high-fidelity compared to low-fidelity settings.However, it also appears that a clear definition of what is meant by high vs. low fidelity is unclear [26–29, 31]. Whereas Isssenberg et al [28] found that high fidelity combined with feedback. would have a positive impact on learning. These studies [26–30] describe the teacher's perspective on fidelity and engagement in learning whensimulating, while our study explored the participants’ perspective. Our findings reveal that participants sometimes attribute their performance gaps to the unfamiliarity of OSS settings. This correlates with Sørensen et al. [32], where participants attribute flaws to the OSS setting but believe these would mitigate over time. Our study shows that the feeling of self-efficacy while performing ISS is higher than when performing OSS, which aligns with other studies in the literature [33, 34].

### Psychological safety

Our study suggests that PS is influenced by both facilitators' and participants' ability to be polite, and that facilitating a learning environment without judgement is crucial for effective learning. This correlates with other research in the field [35–38]. We found that participants perceive the team members' behaviour during the simulation training as crucial to PS. This finding is supported by Edmundson et al. [35], who describe how sharing, creating, and speaking up are key factors to learning. Rudolph et al. [38] show that faculty's behaviour influences PS. However, Rudolph et al. [39] did not address how team behaviour may affect learning. Kostovich et al. [24] found that faculty members try to perceive learners being uncomfortable and intervene to secure a PS learning environment; to some extent, this finding supports our results. However, this is viewed from the faculty’s perspective while our study looked at the simulation participant perspective. Jeffries et al. [40] found that the best enabler of PS was a joint responsibility between the facilitator and students, meaning that the behaviour of both students and the facilitator influence PS. This theme is reiterated in our study as well.

Our findings indicate that the absence of cross-training has a positive influence on PS. Other authors found that people have a strong preference for training in their habitual roles [10, 41]. Thus Klenke-Borgmann et al. [41] noted that some participants perceived it to be contributing to learning to cross train. We found that PS increases when individuals are familiar with their training environment and their roles, resonating with Vygotsky’s Zone of Proximal Development [42]. This suggests that when learners are overwhelmed, it reduces the effectiveness of learning, which aligns with Reedy et al.'s [43] description of cognitive overload and its influence on learning. The team's prior knowledge could influence PS both positively and negatively. This finding correlates with various studies [37, 44]. Henrico et al. [37] describe how the facilitator, as well as the internal and external hierarchies within both the team and the facilitator, influences PS. Furthermore, Lackie et al. [44] found that one-third of all studies demonstrated that power dynamics and hierarchy influenced PS. Within the Danish society workplace hierarchy is among the lowest globally [45]. This may mitigate the effect. Nevertheless, some participants appeared to have pre-existing perceptions about the PS within the department, influencing how they perceived PS during simulation and debriefing. This correlates with Abildgren et al.'s [46] findings showing that participants would take their prior perception of PS in their department into the simulation-based training experience. It is expected that some healthcare professionals collaborate more harmoniously than others. Therefore, we are uncertain whether the feeling of compromised PS can be eliminated entirely. The extent to which this may have influenced our results is uncertain. Nevertheless, a study further exploring this theme would be highly valuable.

In our study, multiple participants state that feeling anxious during the simulation training is healthy and educational as this is how they would feel in real life. This aligns with other studies [42, 47], which suggest that effective learning requires an optimal level of disturbance -not too much, not too little. Furthermore, it correlates with other findings [44], which describe anxiety as a learning experience if it is conducted within an environment with high level of PS. This finding adds to the nuanced understanding of PS in simulation training. Avoidance of cross-training, observers in the room and training in familiar environments are all specific to ISS and cannot entirely be translated to OSS.

### Transfer

Our research indicates that transfer is perceived by participants to occur more frequently in ISS without cross-training. The data reveals that participants training in their habitual role in ISS, apply their learning to their clinical workplace effectively due to consistent environments, teams and high fidelity. Our data further suggest that technical skills are more readily transferred, while social and cognitive skills (SCOPE) are transferred to a lesser extent. This correlates with several studies in the literature [46, 48]. In comparison, Wahlgren et al. [48] showed that a higher degree of integration of the learning environment, acceptance from the department leadership and a greater number of colleagues participating in a course increase the possibility of transfer. Abildgren et al. [46] demonstrated that participants in ISS had a perception of a higher degree of transfer of technical skills than those in SCOPE. We found that organisational transfer can also occur. In Denmark there is not always an assigned anaesthetist to the operating room if the procedure is strictly local anaesthesia. In our department following an incident of anaphylaxis during an ISS, they began assigning an anaesthetist to the operating room daily in case of emergency. The organisational transfer of knowledge is underpinned by several authors [26, 46, 48]. Our findings suggest that transfer occurs on all levels more likely in ISS. Freeman et al. [49] found that novice debriefers would tend to explore the technical aspects of the simulation, whereas more experienced debriefers would focus on SCOPE. We suggest that there might be a correlation between your own colleagues and avoiding subjects within SCOPE. To some, exploring SCOPE may may feel like discussing your personality. This area needs further investigation.

### Cross-training

Our study reveals mixed perception to cross training. The participants with lesser years of post graduate experience tend to perceive cross training as a negative contributer to learning, where as participants with more years of expereince tend to percieve it positiely. This finding aligns with previous research by several authors [11]; notably, Hedges et al. [11] study deliberately employed cross-training among healthcare personnel. Although Hedges et al.’s participants were undergraduate students, this study provides the closest parallel to our results from the interviews. Hedges et al. [11] demonstrated that pharmacy and medical students achieved a greater understanding of each other's workloads following cross-training on specific tasks. Furthermore, Moore et al. [14] showed that a small team of nurses and technicians would achieve a higher degree of team perception after participating in simulation using cross-training. Our study found that more experienced individuals generally have a more positive attitude towards cross-training and that when cross-training is used, most would prefer a role less advanced than their current clinical position, maybe due to less cognitive load. However, it is reasonable to assume that increased experience leads to greater self-efficacy and a higher willingness to train outside one's comfort zone. When using cross-training, awareness of the possibility of cross-training having a negative impact on PS is important. We have been unable to find direct evidence to support or reject this, but it harmonises with the view of the zone of proximal development and the cognitive load theory [42, 43].

### Weaknesses and limitations

This study was conducted in a single unit, examining participants' perceptions on simulation training. It explored their view on cross-training, ISS, and learning experiences beyond OSS. The generalisability, therefore, should be done with caution [50]. Hence, external validity is not given. Furthermore, the first author was both a senior doctor in the department and the lead of the ISS programme, which could potentially influence participants’ answers [51]. This potential bias is known as the Hawthorne effect [52]. To increase the reliability of the data, field notes, audio recordings of debriefings, and interviews were used. When performing interviews, every participant was informed that their participation was voluntary and would have no influence on their future work in the department. Furthermore, researchers would code some of the interviews anonymously compared to the others' codes to increase reliability [53]. Selection bias could also occur [54]. Coding could potentially have influenced the results, as the interviewer and the coder were the same person, making anonymity for the interviewees impossible. The author’s prior perceptions of the subject may also have contributed to the interpretation of the data.

## Conclusion

What are the significant factors that help people learn during ISS? Key factors include team dynamics, timing, and environmental familiarity. We found that fidelity is perceived as higher, not only due to the setting or equipment, but due to how participants engage in their actual work context. Lack of cross-training in our setting also enhances this perceived realism. Furthermore, multiple factors influence PS. This study found that the absence of cross-training and observers has a positive effect on PS. Familiarity with the environment appears to positively influence PS, whereas training in one's own department with present colleagues yields a bilateral perception, showing both positive and negative effects. Learners perceived transfer as more likely during ISS. It was found that participants in generic roles increased both the fidelity and depth of reflection during debriefing.

How do participants perceive the influence of cross-training on learning? Cross-training had mixed effects; some participants felt it hindered learning, while others believed it promoted reflection on their roles. It could negatively impact perceived PS, especially among those with less postgraduate experience. To our knowledge, this is the first study to examine these effects from the participants' perspective.

This study extends existing simulation literature by demonstrating how learners’ perceptions of authenticity, psychological safety, and role alignment shape the educational impact of in situ simulation and cross-training

## Data Availability

The datasets used and analysed in the current study are available from the corresponding author on reasonable request.

## Abbreviations

ISS: In situ simulation – meaning simulation within the clinical environment of the hospital
OSS: Off-Site simulation – meaning simulation within a dedicated simulation facility outside the clinical part of the hospital
CT: Cross training
PS: Psychological safety – meaning how safe it is in each situation for the persons to speak up and express their opinion
SCOPE: Social and Cognitive Skills
I: Interview
